# Sea Lions Develop Human-like Vernix Caseosa Delivering Branched Fats and Squalene to the GI Tract

**DOI:** 10.1038/s41598-018-25871-1

**Published:** 2018-05-10

**Authors:** Dong Hao Wang, Rinat Ran-Ressler, Judy St Leger, Erika Nilson, Lauren Palmer, Richard Collins, J. Thomas Brenna

**Affiliations:** 1 Division of Nutritional Sciences, Cornell University, Ithaca, NY 14853 USA; 2 Department of Food Science and Technology, Cornell University, Ithaca, NY 14853 USA; 3 Seaworld, San Diego, CA 92109 USA; 4 The Marine Mammal Care Center at Fort MacArthur, 3601 S, Gaffey St #8, San Pedro, CA 90731 USA; 5 Studiecentrum Antropologie, Mechelbaan 338, 2580 Putte, Belgium; 6 Dell Pediatric Research Institute, Departments of Pediatrics, of Nutrition, and of Chemistry, University of Texas at Austin, 1400 Barbara Jordan Blvd, Austin, TX 78723 USA

## Abstract

Vernix caseosa, the white waxy coating found on newborn human skin, is thought to be a uniquely human substance. Its signature characteristic is exceptional richness in saturated branched chain fatty acids (BCFA) and squalene. Vernix particles sloughed from the skin suspended in amniotic fluid are swallowed by the human fetus, depositing BCFA/squalene throughout the gastrointestinal (GI) tract, thereby establishing a unique microbial niche that influences development of nascent microbiota. Here we show that late-term California sea lion (*Zalophus californianus*) fetuses have true vernix caseosa, delivering BCFA and squalene to the fetal GI tract thereby recapitulating the human fetal gut microbial niche. These are the first data demonstrating the production of true vernix caseosa in a species other than *Homo sapiens*. Its presence in a marine mammal supports the hypothesis of an aquatic habituation period in the evolution of modern humans.

## Introduction

Vernix caseosa (“cheesy varnish”) is the white, fat laden material found on the skin of human newborns, long thought to be unique to humans^1^. Vernix is synthesized by the fetal skin sebaceous glands, and is approximately half lipid on a dry matter basis, including shed fetal corneocytes^2^. The fatty acyl chains of the lipid component is unique among human substances, containing about 30% saturated monomethyl branched chain fatty acids (BCFA) with branching near the terminal end of the acyl chains^3^. Vernix BCFA characteristically consist of a broad distribution of acyl chain lengths from about C_11_ to C_26_, similar to other BCFA distributions originating in the skin such as lanolin (sheep wool fat). Milk is another major mammalian substance with significant concentrations of monomethyl BCFA, having a much narrower range of chain lengths. Bovine^4^, goat^5^, or yak milk BCFA have chain lengths from about C_14_ to C_18_ biosynthesized by rumen bacteria, and present at about 2–3% of total fatty acids. Human breast milk fat contains 1–2%^6,7^. The distribution of BCFA chain lengths serves as a marker of their origin: BCFA with chain length distributions within range of C_14_-C_18_ originate in milks, while distributions outside this range originate in skin.

Squalene is a major component of human surface lipids, but in contrast to BCFA is rare in other species. Squalene comprises about 12% surface lipids (sebum and meibum) in human adults^8,9^ and is also a component of human vernix caseosa, at about 9%^10^. Apart from humans, squalene has been reported in the sebum of only four mammals of more than 60 species analyzed to date, all living in aquatic or wet environments^11–13^.

The biological role of vernix is not well understood; most hypotheses are related to skin function. Vernix is thought to be a barrier to water loss, assists in neonatal temperature regulation as an insulating layer, and supports innate immunity including antibacterial activity^14^. A complementary hypothesis is that vernix serves a nutritional role as a non-fermentable prebiotic, and alters the physiology of constituent microorganisms. In the last third of gestation, solid vernix particles slough off the skin where they cause amniotic fluid to become increasingly turbid, reaching a maximum particle density at about week 37 of the normal 40 week human gestation. Through the last trimester, human fetuses actively swallow hundreds of milliliters of amniotic fluid daily, and with it vernix particles. In this respect vernix caseosa can be considered the first solid meal of humans. Vernix BCFA are found in meconium, the first fecal excretion of newborns, with chain lengths corresponding to higher molecular masses^3^. BCFA are therefore at high concentration throughout the newborn GI tract as the first organisms are arriving to colonize the gut. BCFA are major constituents of the membranes of many microorganisms, reaching 95% of fatty acyl chains and are particularly rich in most species in the *bacilli* genus. In membranes, BCFA serve similar biophysical functions as *cis* unsaturated double bonds, lowering phase transition temperatures while avoiding vulnerability of double bonds and their active allylic sites to oxygen attack^15^. In a newborn rat pup model of necrotizing enterocolitis (NEC), BCFA treatment shifted the distribution of the nascent microbiota toward organisms known to use BCFA in their membranes while reducing the incidence of NEC by >50%^16^. Recent evidence shows that BCFA are taken up, incorporated into human enterocyte membrane phospholipids, and confer anti-inflammatory activity against lipopolysaccharide-induced inflammation^17,18^. BCFA are also known *in vitro* to reduce motility, and presumably virulence, of the pathogen *Pseudomonas aeruginosa*^19^. As a component of surface skin lipids, squalene acts as an emollient and antioxidant and has antitumor effects^20^.

**Figure 1:**
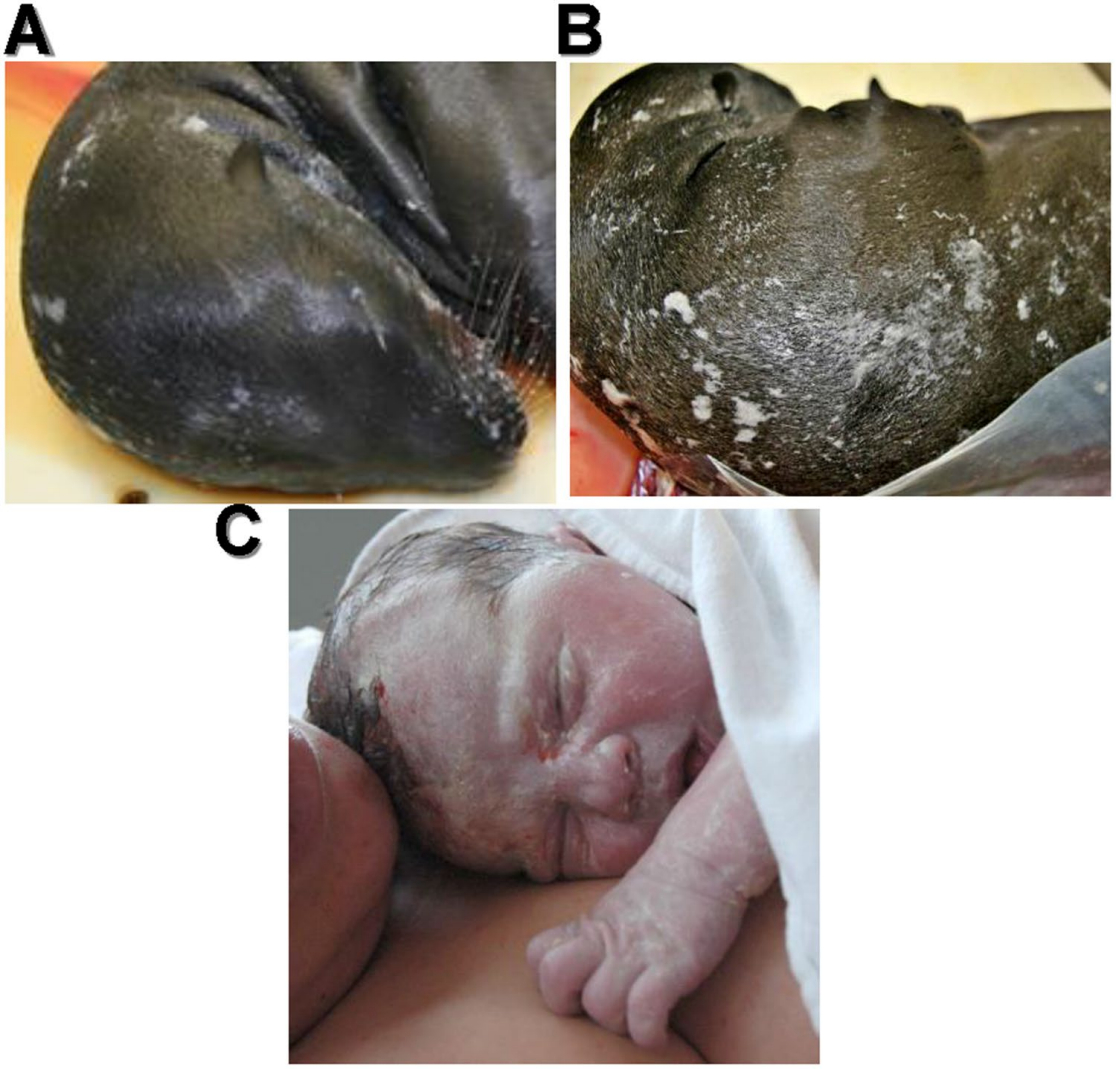
Vernix caseosa visible on a late-term California sea lion fetus and a human newborn. (**A**) The sea lion has white debris evident on the whiskers, eyebrows, head, and neck, and (**B**) along the back. (**C**) A human newborn seconds old, with vernix caseosa on most of the skin.

In May of 2013, an algal bloom caused a domoic acid event off the coast of Southern California resulting in late-term abortions and maternal death of wild California sea lions. We examined fetuses from these animals at a stranded animal facility in San Diego, California. Figure 1 is a late-term California sea lion (*Zalophus californianus*) fetus collected from a deceased pregnant female with a human newborn for comparison. The sea lion back shows a patchy white film similar to the appearance of vernix caseosa on human newborns. The whiskers in particular display small clumps of white material notable at the base. The clumping is similar to that on eyebrows and lanugo, the fine fetal body hair, of humans.

We hypothesized that this material is the sea lion equivalent of human vernix caseosa, synthesized in the skin and swallowed as amniotic fluid borne particles to appear in the gastrointestinal (GI) tract. Because a broad chain length distribution of BCFA and squalene are the signature characteristics of surface lipids, we analyzed surface material, fetal GI tract contents, and serum for comparison to existing data on the analogous human substances.

## Results

**Figure 2:**
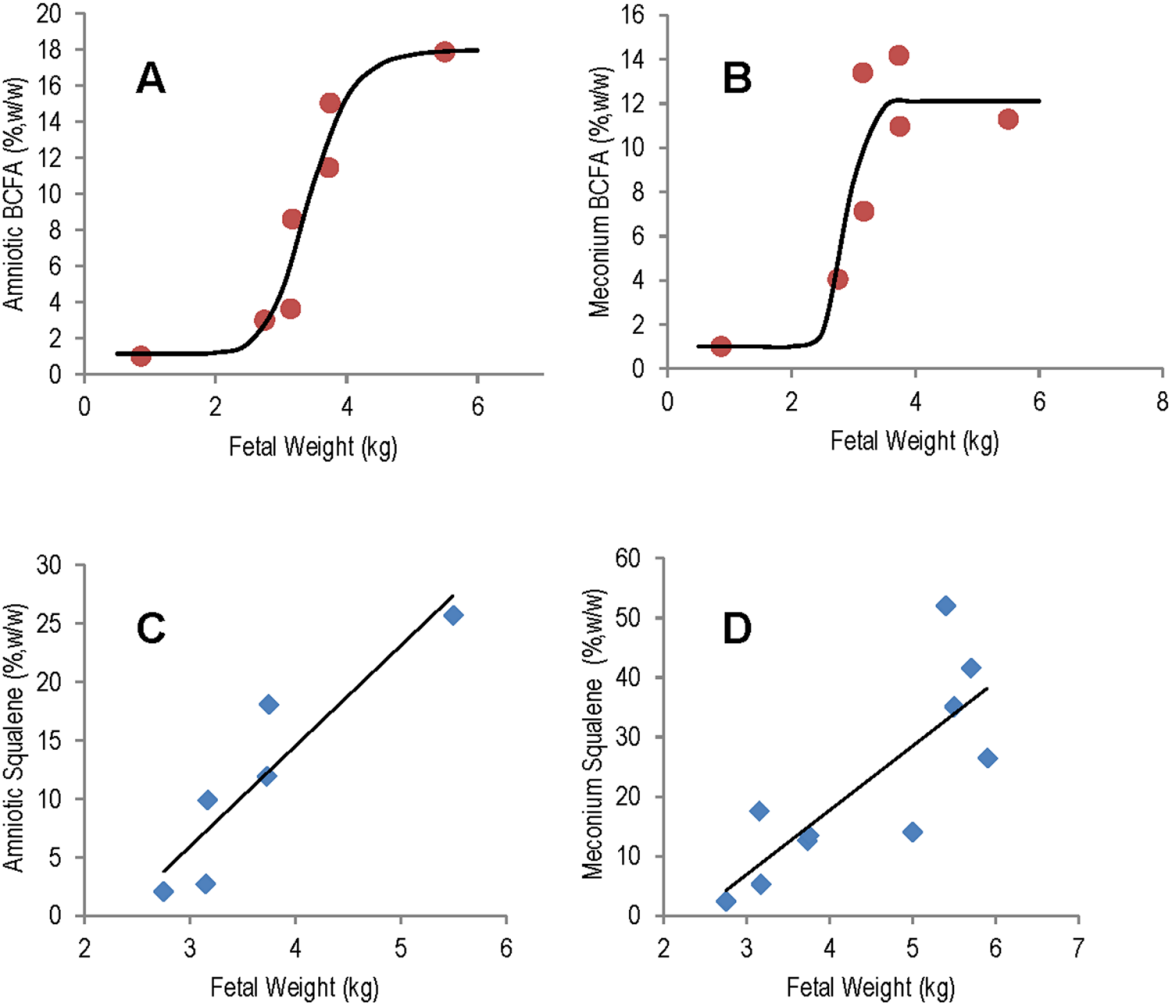
BCFA and squalene in amniotic fluid and meconium increase with gestational weight gain. (**A**)The sigmoidal shapes are consistent with development of vernix in the last half of gestation, as in humans. Meconium only was available from the 0.86 kg early-gestation sea lion fetus and is plotted for amniotic fluid modeling. B = 18.0 + (1.15–18.0)/(1 + (w/3.42)^10.6^); r^2^ = 0.93; p = 0.04. B is total BCFA, and w is fetal weight. (**B**) Meconium BCFA. B = 12.1 + (0.997–12.1)/(1 + (w/2.89)^19.1^); r^2^ = 0.81, p = 0.18. (**C**) Squalene in amniotic fluid shows a linear growth trend. S = 8.59w − 19.8; r^2^ = 0.84; p < 0.001. (**D**) Squalene in meconium. S = 10.8w − 25.4; r^2^ = 0.64; p < 0.001.

Samples were collected at necropsy from six sea lion fetuses, two males and four females, of weights 2.75 to 5.50 kg and lengths 46 to 67 cm. The percentage of BCFA found in total lipids of amniotic fluid and meconium increased sigmoidally with fetal weights (Fig. 2A,B). Our regression model indicated that early fetal BCFA were about 1% in both amniotic fluid and meconium and rose dramatically throughout the amnion-gut environment beyond a fetal weight of 2.5 kg. The BCFA accumulation was most rapid by about 4–5 kg in fetal weight and then plateaued near parturition. In the heaviest fetus (late term), amniotic lipid BCFA was 17.9%, while meconium lipid was 11.3%. The pattern is similar to that in human fetuses, which begin to release BCFA-containing vernix particles into the amniotic fluid until the second half of gestation; human meconium BCFA is 17.5%, very similar to that of the heaviest fetal sea lion. Squalene content in the same sample types increased linearly with gestational weight gain (Fig. 2C,D). From the linear regression model of both amniotic fluid and meconium, we extrapolate that the production of squalene starts at a weight of 2.3 kg for California sea lion fetuses.

**Figure 3:**
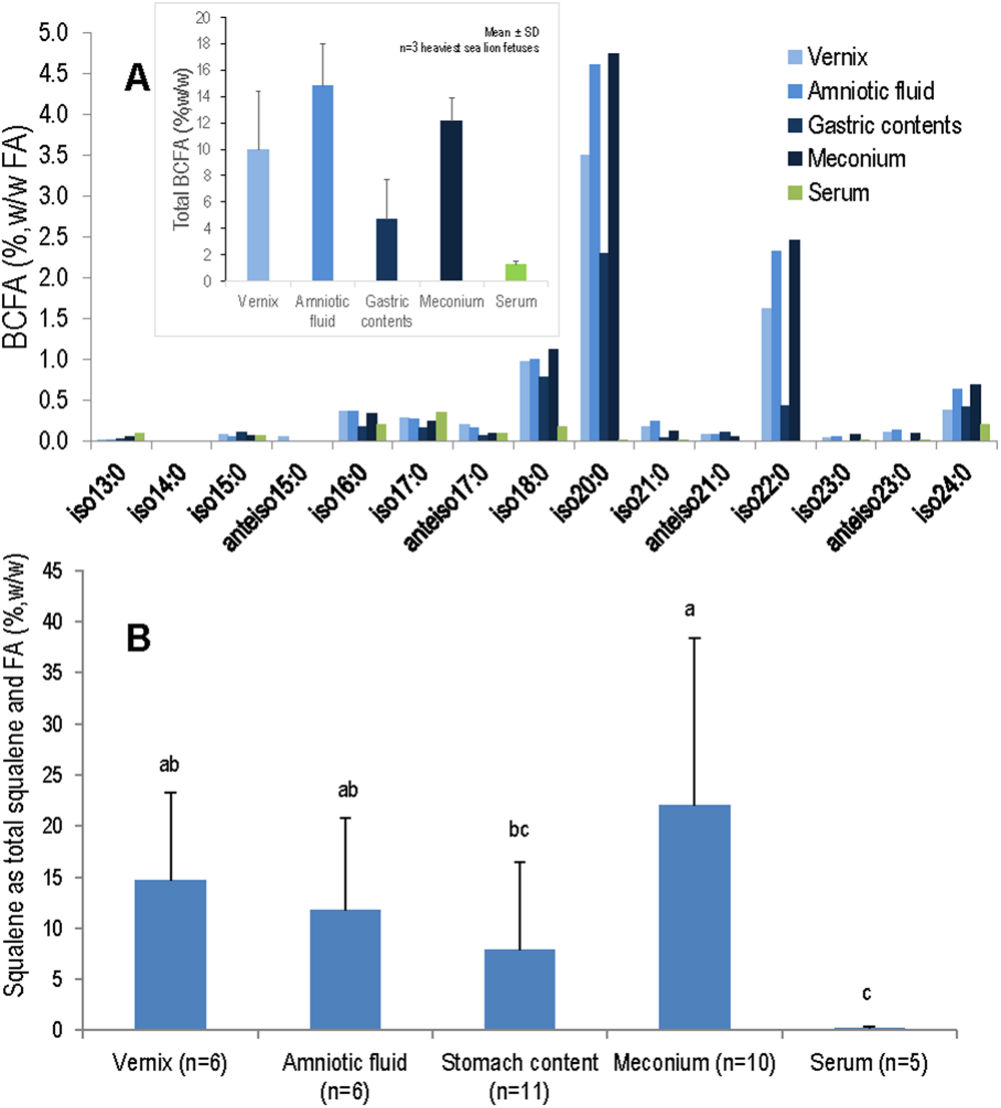
California sea lion fetal BCFA/squalene in vernix, amniotic fluid, gastric content, meconium, and serum (n = 6). (**A**) *iso*-BCFA C_18_-_24_ dominate the distribution, similar to human BCFA, and unlike milkfat distributions C_14_-_18_ (Supplementary Fig. 2). Serum BCFA distribution is dissimilar, C_16_-_18,24_, showing BCFA are only selectively transported to the circulation. Technical CV averages 3%. Inset: Summed BCFA in each substance for the three heaviest fetuses average about 10% except for serum. (**B**) Squalene is high in all substances except serum. Different letters signify a significant difference.

BCFA in vernix, amniotic fluid, gastric contents, and meconium in sea lion fetuses shows a distribution of chain lengths from C_11_ to C_24_, and consisting of isomers terminating in propan-2-yl (*iso*) and butan-2-yl (*anteiso*) groups (Fig. 3A). This broad distribution is indicative of origin on the skin and not milk. Similar to human vernix and meconium (Fig. S1A), sea lion vernix and meconium BCFA are rich in the *iso* configuration, showing a dominant pattern suggesting two carbon elongation in the sequence *iso*-16:0 → *iso*-18:0 → *iso*-20:0 → *iso*-22:0 → *iso*-24:0. *iso*-20:0 was at highest concentration in all pools, ranging from 2.3% in stomach contents to 4.7% in meconium. *iso*-20:0 and *iso*-22:0 comprised 5.1% and 7.2% of sea lion vernix and meconium, respectively, similar to human vernix and meconium of 3.1% and 6.5% *iso*-20:0 and *iso*-22:0, respectively. In contrast, bovine milk, another major BCFA containing substance, has BCFA from C_14_-C_18_ (Fig. S1B) and milks of Atlantic grey seals, Antarctic fur seals, stellar sea lions, and New Zealand sea lions have BCFA from C_15_-C_18_. Fetal sea lion serum BCFA were also detected, however in contrast to the other substances, the maximal BCFA was *iso*-17:0 at 0.35%, again consistent with low BCFA detected in human plasma^21^. Levels of total BCFA for the three heaviest fetuses range from 4.7 to 14.8% for gastric contents and amniotic fluid, respectively, while serum total BCFA was 1.3% (Fig. 3, inset).

We consider squalene as a percentage of total fatty acids as a convenient normalization factor. Squalene reaches a peak of 22%,w/w (of total fatty acids) in meconium, while it is never more than at trace levels in serum. Statistical analysis shows that serum squalene is significantly lower than meconium, vernix, and amniotic fluid. Meconium squalene is significantly higher than squalene content in stomach fluid. Mean squalene values for amniotic fluid and stomach content are 12% and 8%, respectively. Of the four heaviest fetuses, meconium squalene was as high as 40%. Amniotic fluid squalene was about 25% in the pup whose weight (5.5 kg) was slightly below the normal term weight of 6 kg. Vernix squalene was 15% (Fig. 3B), similar to its level in human vernix (~9%)^10^.

## Discussion

Our data show for the first time that vernix caseosa is not unique to *homo sapiens* but also occurs in at least one species of marine mammal, the California sea lion. The patches of skin surface resident BCFA and squalene in amniotic fluid, stomach contents, and meconium strongly implies that vernix is playing a similar nutritional role in the California sea lion as in humans. Vernix caseosa functions are not universally agreed upon though the obvious possibilities that it plays a protective role on the skin against infection and excess water loss has been long investigated. Naked/nearly hairless mammals such as pigs have no vernix, nor do rodents who are born with no hair, thus vernix cannot be a requirement for hairlessness. We can speculate that BCFA/squalene-rich vernix would favor the development of a commensal microbiota on the skin as well as in the GI tract. Mammals born into a wet but not submerged environment such as the shoreline may be more vulnerable to microbial inoculation carrying potential pathogens from the environment to the skin and the GI tract than, for instance, mammals born on the savannas. Once breathing is established, the first behavior of mammals is to nurse, immediately flooding the GI tract with living cell and antibody rich milk and microbiota from the nipple to augment microbes entering the mouth and nose from the birth canal. Mammals born into a dry savanna environment who immediately stand and nurse may be less exposed to pathogens than those born into a microbe-rich wet environment.

Squalene has long been known to be a major component of human surface lipids. Of at least 60 mammals reported to date, squalene has been found in the sebum of only four other mammals: the aquatic beaver and otter, the nocturnal arboreal rainforest dwelling kinkajou, and the mole, known as a semi-aquatic animal partially adapted to underwater foraging^22^. Early authors investigating sebum lipid classes observed that squalene is only found in animals that inhabit a “damp environment” suggesting a function for squalene in skin lipids of mammals whose surface is often wet^11^. California sea lions are now the sixth species.

Squalene is a hexaene polyunsaturated, multiply branched isoprenoid hydrocarbon that is an intermediate in the endogenous synthesis of cholesterol; the large amounts in vernix may serve as an efficient precursor for cholesterol in the GI or post-absorption. Squalene double bonds are all in the trans (E) configuration and separated by an ethylene group, thus the molecule does not possess the more highly activated *bis* allylic –CH2– of most polyunsaturated fatty acids. Squalene is an active singlet oxygen (^1^O_2_) scavenger, the most active among skin lipids and is protective against radiation induced (e.g. UV) ^1^O_2_ production in the lipids of the tear film. Neutrophils and macrophages are known to produce ^1^O_2_ presumably for bacterial killing. Inflammation-related human GI diseases including NEC are associated with oxidative stress; squalene entering the GI tract via vernix plausibly is an endogenous defense^23^. Finally, squalene may play a role in selection of surface microbiome/mycobiome since it is a major component of the lipids of a diverse range of microorganisms including fungi, possibly favoring organisms by supplying an essential metabolite from the environment rather than expending the energy needed for synthesis^24^.

Sir David Attenborough raises the issue of vernix as one of the many human traits parallel to aquatic adaptation of marine mammals^25,26^. The role of aquatic resources in human evolution is controversial, inspired recently by energetic considerations for supporting human brain growth^27^. The concept of brain-selective nutrients including omega-3 docosahexaenoic acid and many minerals (iodine, iron, copper, zinc, selenium) plentiful at land-water interface but scarce on arid savannahs has been used to support the concept of a semi-aquatic phase in the rapid expansion of the human brain^28,29^. The unique observation of BCFA/squalene in the GI tract in newborns suggests an advantage to shifting properties of the nascent microbiota in a way shared by marine mammals and humans, and possibly a metabolic parallel in enterocytes or other intestinal function.

## Materials and Methods

### Ethical approval

This work was authorized under the US Marine Mammal Protection Act; samples were collected under National Marine Fisheries Scientific Research Permit No. 932-1489-00. All experiments were performed in accordance with relevant guidelines and regulations.

### Sample collection

Vernix, amniotic fluid, gastric content, meconium, and serum of late to full-term were collected during necropsies from six sea lion pregnant animal/fetal pairs. Additional meconium and gastric contents were obtained from other pairs in the same manner for squalene analyses. All tissues and fluids were stored in dry ice or at −80 °C until analysis.

### Fatty acid analysis

Total lipids were extracted from meconium, amniotic fluid, and gastric contents according to a routine method as described in detail elsewhere^4^. Briefly, samples (~100 mg) were extracted and methylated according to a modified one-step hydrolysis procedure^30^. Heneicosanoic acid (21:0) was used as the internal standard. Fatty acid methyl esters (FAME) were identified and analyzed quantitatively using an HP 5890 gas chromatograph equipped with a BPX-70 capillary column (25 m × 0·22 mm × 0·25 mm; SGE) with H_2_ carrier gas and flame ionization detector. A FAME mixture of equal weight (68A; Nu-Chek Prep, Inc.) was used to calculate response factors and six BCFA were used as authentic reference standards (*iso*-14:0, *anteiso*-15:0, *iso*-16:0, *anteiso*-17:0, *iso*-18:0, and *iso*-20:0; Larodan Fine Chemicals AB). FAME identities were determined by electron ionization MS, chemical ionization, and electron ionization – tandem MS, as described previously using a Varian Star 3400 GC coupled to a Varian Saturn 2000 ion trap MS^31^.

## Electronic supplementary material

Supplementary Information
