# Supplementary material for: Sea Lions Develop Human-like Vernix Caseosa Delivering Branched Fats and Squalene to the GI Tract
Source: Sci Rep. 2018 May 10;8:7478. doi: 10.1038/s41598-018-25871-1 (PMC5945841; doi:10.1038/s41598-018-25871-1)
Supplement: Supplementary file 1 — Supplementary Information [file 41598_2018_25871_MOESM1_ESM.docx]

Sea Lions Develop Human-like Vernix Caseosa Delivering Branched Fats and Squalene to the GI Tract

Dong Hao Wang, Rinat Ran-Ressler, Judy St. Leger, Erika Nilson, Lauren Palmer, Richard Collins, J. Thomas Brenna

Supplementary Materials

Figure S1

Table S1

Figure S1. (A) Human vernix and meconium BCFA distributions (1). (B) Cow milkfat BCFA distribution (2).

**A**

**B**

Table S1. Fatty acid composition of vernix, amniotic fluid, gastric contents, meconium and serum from late-term California sea lion fetuses.

|  | Vernix | Amniotic fluid | Gastric content | Meconium | Serum |
| --- | --- | --- | --- | --- | --- |
| *iso*-13:0 | 0.01 | 0.02 | 0.03 | 0.05 | 0.09 |
| 13:0 | 0.01 | 0.00 | 0.01 | 0.02 | 0.01 |
| *iso*-14:0 | 0.00 | 0.00 | 0.00 | 0.01 | 0.00 |
| 14:0 | 1.96 | 1.62 | 1.13 | 0.91 | 1.43 |
| *iso*-15:0 | 0.08 | 0.06 | 0.11 | 0.06 | 0.08 |
| 14:1 | 0.08 | 0.06 | 0.06 | 0.06 | 0.15 |
| *anteiso*-15:0 | 0.06 | 0.00 | 0.00 | 0.00 | 0.00 |
| 15:0 | 1.04 | 0.63 | 0.50 | 0.68 | 0.64 |
| *iso*-16:0 | 0.36 | 0.37 | 0.18 | 0.34 | 0.21 |
| 16:0 | 45.26 | 36.42 | 28.92 | 31.76 | 34.47 |
| 16:1n-9 | 1.82 | 1.64 | 1.17 | 0.54 | 1.13 |
| 16:1n-7 | 2.13 | 1.89 | 2.83 | 1.21 | 4.71 |
| *iso*-17:0 | 0.28 | 0.27 | 0.16 | 0.25 | 0.35 |
| *anteiso*-17:0 | 0.20 | 0.16 | 0.06 | 0.09 | 0.10 |
| 17:1 | 0.11 | 0.05 | 0.03 | 0.06 | 0.27 |
| 17:0 | 0.53 | 0.64 | 0.62 | 0.88 | 0.40 |
| 17:1n-8 | 0.26 | 0.23 | 0.44 | 0.66 | 0.16 |
| 17:1b | 0.09 | 0.11 | 0.14 | 0.04 | 0.19 |
| *iso*-18:0 | 0.97 | 1.00 | 0.79 | 1.12 | 0.18 |
| 18:0 | 8.83 | 8.90 | 13.06 | 17.64 | 7.96 |
| 18:1 isomers | 13.25 | 18.09 | 21.67 | 13.06 | 25.54 |
| 19:0 branched | 0.11 | 0.10 | 0.11 | 0.13 | 0.23 |
| 18:2 | 0.11 | 0.09 | 0.09 | 0.06 | 0.03 |
| 18:2n-6 | 0.44 | 0.38 | 0.63 | 0.40 | 0.43 |
| 19:0 | 0.89 | 0.66 | 0.65 | 0.92 | 0.37 |
| 18:3n-6 | 0.08 | 0.18 | 0.08 | 0.16 | 0.12 |
| 19:1 isomers | 0.12 | 0.09 | 0.08 | 0.09 | 0.10 |
| *iso*-20:0 | 3.50 | 4.60 | 2.30 | 4.74 | 0.01 |
| 20:1 | 0.06 | 0.16 | 0.04 | 0.08 | 0.00 |
| 20:0 | 1.44 | 1.70 | 0.92 | 2.77 | 0.10 |
| 20:1n-9 | 0.96 | 1.15 | 0.62 | 1.21 | 0.49 |
| 20:1n-7 | 0.36 | 0.40 | 0.23 | 0.27 | 0.23 |
| *iso*-21:0 | 0.18 | 0.24 | 0.04 | 0.12 | 0.02 |
| 20:2n-9 | 0.40 | 0.37 | 0.16 | 0.23 | 0.26 |
| *anteiso*-21:0 | 0.08 | 0.08 | 0.11 | 0.06 | 0.00 |
| 20:2n-6 | 0.68 | 0.81 | 0.32 | 0.37 | 0.51 |
| 21:0+20:3n-9 | 0.42 | 0.60 | 0.37 | 0.40 | 0.32 |
| 20:3n-6 | 0.10 | 0.14 | 0.19 | 0.18 | 0.12 |
| *iso*-22:0 | 1.62 | 2.32 | 0.44 | 2.46 | 0.00 |
| 20:4n-6 | 3.59 | 5.13 | 11.20 | 4.58 | 10.32 |
| 22:1 | 0.08 | 0.09 | 0.05 | 0.05 | 0.00 |
| 22:0 | 0.75 | 0.79 | 0.47 | 1.68 | 0.03 |
| 22:1 isomers | 1.09 | 1.63 | 1.02 | 0.60 | 1.18 |
| *iso*-23:0 | 0.05 | 0.06 | 0.00 | 0.08 | 0.01 |
| 20:5n-3 | 0.12 | 0.10 | 0.22 | 0.04 | 0.31 |
| *anteiso*-23:0 | 0.11 | 0.13 | 0.01 | 0.09 | 0.01 |
| *iso*-24:0 | 0.38 | 0.63 | 0.42 | 0.70 | 0.21 |
| 22:4n-6 | 0.19 | 0.27 | 0.32 | 0.52 | 0.23 |
| 24:0 | 0.82 | 0.76 | 0.49 | 1.75 | 0.05 |
| 24:1 | 0.49 | 0.57 | 0.47 | 0.87 | 0.61 |
| 22:5n-3 | 1.58 | 1.69 | 1.92 | 2.11 | 3.13 |
| 22:6n-3 | 1.21 | 1.51 | 2.68 | 1.98 | 2.33 |
| 26:0 | 0.19 | 0.14 | 0.34 | 0.23 | 0.00 |
| 26:1 isomers | 0.35 | 0.27 | 1.02 | 0.36 | 0.00 |
| 28:1a | 0.00 | 0.00 | 0.00 | 0.07 | 0.00 |
| 28:1b | 0.00 | 0.00 | 0.00 | 0.08 | 0.00 |

a,b concentrations are isomers of concentration below that required to identify structure.

Supplementary references

1. Ran-Ressler RR, Devapatla S, Lawrence P, & Brenna JT (2008) Branched chain fatty acids are constituents of the normal healthy newborn gastrointestinal tract. *Pediatr Res* 64(6):605-609.

2. Ran-Ressler RR*, et al.* (2011) Branched chain fatty acid content of United States retail cow's milk and implications for dietary intake. *Lipids* 46(7):569-576.
